# Garlic (*Allium sativum*) and mushroom (*Agaricus bisporus*) powder: Investigation of performance, immune organs and humoural and cellular immune response in broilers

**DOI:** 10.1002/vms3.1367

**Published:** 2024-02-15

**Authors:** Hadi Noruzi, Fatemeh Aziz‐Aliabadi

**Affiliations:** ^1^ Department of Animal Science, Faculty of Agriculture Ferdowsi University of Mashhad Mashhad Iran

**Keywords:** broilers, garlic, immune response, mushroom, Newcastle disease, performance

## Abstract

**Background:**

Alternatives to antibiotics have been suggested by banning their use in the poultry industry. Garlic and mushroom are two important phytobiotic compounds in poultry nutrition.

**Objectives:**

This study was conducted to evaluate the effects of supplementing diets with garlic and mushroom powder (MP) on growth performance, humoural and cellular immunity, and white blood cell counts of broiler chickens.

**Methods:**

Five hundred and seventy‐six 1‐day‐old male broiler chickens (Ross 308) were assigned to 8 treatments with 6 replications (12 birds per replication) based on a completely randomized design in a factorial arrangement of 4 × 2 with 4 levels of garlic powder (GP; 0.00%, 0.50%, 1.00%, and 1.50%) and two levels of MP (0.00% and 1.00%).

**Results:**

No significant effects of GP and MP on the growth performance and cutaneous basophil hypersensitivity were observed (*p* > 0.05). According to the regression equation, with increasing levels of GP in the diets, the relative weight of the bursa of Fabricius and thymus increased (*p* < 0.05). The effect of increasing levels of GP on the Newcastle disease virus (NDV) titre was greater in the absence of MP (*p* < 0.05). With increasing levels of GP in the diets, the percentages of lymphocytes and heterophils to lymphocytes ratio increased and reduced, respectively (*p* < 0.05).

**Conclusions:**

This experiment has revealed that increasing the level of GP improved the immune response of broilers without affecting performance. The effect of increasing the level of GP on the NDV titre was more significant in the absence of MP.

## Graphical abstract

- Five hundred and seventy‐six one‐day‐old Ross 308 broiler chickens were assigned to eight treatments with three levels of garlic powder and two levels of mushroom powder for 42 days.
- Increasing the level of garlic powder ameliorated the broilers’ immune system without affecting the growth performance.
- The effect of increasing the level of garlic on the Newcastle disease virus titre was more in the absence of mushroom.

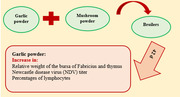

## 1 INTRODUCTION

Because of the ban on antibiotic use, nutritionists have focused on alternatives to antibiotics in the last decade. Recently, many studies have been conducted on substitute natural ingredients, including garlic and mushrooms. Garlic (*Allium sativum*) is generally applied as a stuffing factor for food or as a conventional remedial plant to cure several morbidities (Amagase et al., 2001; Konjufca et al., 1997). The remedial and antioxidant impacts of garlic's flavonoid and organosulphur compounds and their derivatives have been shown on broilers and layers (Chowdhury et al., 2002; Sallam et al., 2004). Garlic also has a powerful antimicrobial effect (Onibi et al., 2009). Allicin, which generally comprises more than two thirds of the total thiosulfinates, is the principal sulphur comprising ingredients in garlic (Rybak et al., 2004). It has been stated that allicin and its derivatives are considered a larvicide and bacteriostatic, which act effectively against gram‐positive or gram‐negative organisms, as well as fungi such as *Candida albicans* and influenza virus (Chang & Cheong, 2008). Various studies support the role of garlic in improving growth performance, meat quality, anti‐cholesterolemic effects and strengthening the immune system in broiler chickens (Aji et al., 2011; Ashayerizadeh et al., 2009; Ayasan, 2011; Gardzielewska et al., 2003; Hanieh et al., 2010). Javandel et al. (2008) reported that groups receiving diets containing less than 2.00% garlic meal had better growth performance and feed conversion ratio (FCR) as compared to the control group. They said that the diets containing 2.00% garlic meal negatively affected birds’ performance.

Mushrooms and their diverse derivatives possess a variety of active compounds like ergothioneine, phenolic antioxidants, variegatic acid and diboviquinone (Dalloul et al., 2006; Dubost et al., 2007; Kasuga et al., 1995). Mushrooms’ effective substances include antioxidant, antibacterial, immune enhancing and stress reduction activities (Dalloul & Lillehoj, 2006). Giannenas et al. (2010) reported the beneficial effect of mushrooms (*Agaricus bisporus*) on the broiler's performance and tissue antioxidant‐protective activity. Several studies recommend garlic and mushroom as advantageous growth boosters for broilers, and most of them have shown positive results. Nevertheless, the differences in the trial situations, poultry health, genetics, characteristics and processing of these compounds have eventuated in some eristic viewpoints. Based on these studies, it can be suggested that the mixture of these substances may have specific benefits and be helpful as an alternative to antibiotics. Therefore, this study aimed to investigate the effects of garlic and mushroom powder (MP) on the growth performance, carcass traits and humoural and cellular immune response of broiler chickens.

## 2 MATERIALS AND METHODS

### 2.1 Garlic and mushroom powder preparation and analysis

Fresh garlic and mushrooms were purchased from a local marketplace (my country). Fresh garlic was hulled, broken into pieces and dehumidified under sunlight at 30–35°C for 1 day (Lim et al., 2006). The dried samples were further dehumidified under 50°C in an oven and finely milled to a powder (Chowdhury et al., 2002). In order to prepare MP, mushrooms were cut into slices with a thickness of 5 mm, dried at 55–60°C and then carefully ground (Kavyani et al., 2014). The dried garlic powder (GP) contained 901.11 g of dry matter/kg, 119.41 g of crude protein/kg, 27.00 g of crude fat/kg, 16.34 g of crude fibre/kg and 31.52 g of crude ash/kg (Association of Official's Analytical Chemists [AOAC], 2005). The dried MP included 913.16 g of dry matter/kg, 125.17 g of crude protein/kg, 41.09 g of crude fat/kg, 64.19 g of crude fibre/kg and 76.13 g of crude ash/kg (Association of Official's Analytical Chemists [AOAC], 2005).

### 2.2 Birds and management

Five hundred and seventy‐six 1‐day‐old male Ross 308 broiler chickens were provided from a native commercial hatchery and reared for 42 days of age. Eight experimental diets (4 × 2) that were fed to the birds during the starter (1–10 days of age), grower (11–24 days of age) and finisher (25–42 days of age) periods included (1) no GP + no MP (control), (2) 0.50% GP + no MP, (3) 1.00% GP + no MP, (4) 1.50% GP + no MP, (5) no GP + 1.00% MP, (6) 0.50% GP + 1.00% MP, (7) 1.00% GP + 1.00% MP and (8) 1.50% GP + 1.00% MP. The components and nutrient composition of diets are presented in Table 1 (National Research Council [NRC], 1994). All diets were formulated based on the Ross 308 nutrition guidelines (Aviagen, 2014). The temperature of the rearing place was set at 32°C in the first 3 days and reduced by 3°C each week to reach 21°C and remained constant until the end of the study. Relative dampness was between 60% and 70%. The first 2 days were set with a 24‐h light schedule followed by 18 h of light and 6 h of darkness. The birds had access to feed and water during the whole experimental period.

**TABLE 1:** Ingredients and nutrient composition of the experimental diets (as‐fed basis).

Ingredients (%)	Starter (1–10 days)	Grower (11–24 days)	Finisher (25–42 days)
Corn	52.39	55.75	61.02
Soybean meal (44%)	36.45	32.67	27.22
Corn gluten meal (62%)	3.00	3.00	3.00
Soybean oil	3.30	4.18	4.72
Limestone	1.08	0.99	0.91
Dicalcium phosphate	1.97	1.75	1.55
Common salt	0.26	0.26	0.26
Vitamin premix^a^	0.25	0.25	0.25
Mineral premix^b^	0.25	0.25	0.25
l‐Lysine HCl	0.37	0.30	0.29
dl‐Methionine	0.35	0.30	0.26
l‐Threonine	0.13	0.10	0.08
Sodium bicarbonate	0.16	0.16	0.16
Choline chloride 60%	0.04	0.04	0.04
Nutrient composition (%)			
Metabolizable energy (kcal/kg)	3000	3100	3200
Crude protein	23.00	21.50	19.50
Calcium	0.96	0.87	0.78
Available phosphorus	0.48	0.43	0.39
Sodium	0.16	0.16	0.16
Lysine	1.44	1.29	1.15
Methionine + cystine	1.08	0.99	0.90
Threonine	0.97	0.88	0.78

^a^ Provided the followings per kg of diet: vitamin A (trans‐retinyl acetate), 12,500 U; vitamin D3 (cholecalciferol), 5000 U; vitamin E (dl‐α‐tocopherol acetate), 80 U; vitamin K (menadione), 3.00 mg; riboflavin, 7.50 mg; pantothenic acid (d‐Ca pantothenate), 17.50 mg; pyridoxine (pyridoxine‐HCl), 4.50 mg; thiamine, 3.00 mg; vitamin B12 (cyanocobalamin), 0.02 mg; biotin, 0.20 mg; folic acid, 2.00 mg; nicotinic acid, 65.00 mg; ethoxyquin (antioxidant), 2.50 mg.

^b^ Provided the following per kg of diet: Fe, 20.00 mg; Zn, 110.00 mg; Mn, 120.00 mg; Cu, 16.00 mg; I, 1.25 mg; Se, 0.30 mg.

### 2.3 Performance and internal organs

At the end of each phase (11, 25 and 42 days of age), the birds of each pen were weighed. The daily weight gain (DWG), daily feed intake and FCR were measured for each pen according to Noruzi et al. (2023)’s method. At the end of the experiment (42 days of age), two birds from each pen were picked out randomly and euthanized by cervical dislocation. Before slaughtering, birds were deprived of feed for 12 h and weighed. The spleen, bursa, thymus and liver were removed, weighed and recorded as the percentage of live weight.

### 2.4 Estimation of humoural immune response

Chickens were vaccinated against Newcastle disease virus (NDV) and avian influenza (AI) on day 28. Then, in order to study the titre of influenza and Newcastle vaccines, blood samples were collected (from two birds in each pen) at 42 days of age, kept until clotting and then centrifuged at 3000 × *g* for 15 min, and the sera samples are stored at −20°C until the determination of antibody titre. The samples from each group were analysed by enzyme‐linked immunosorbent assay kits (IDEXX Laboratories, Inc.).

### 2.5 Cutaneous basophil hypersensitivity

Two birds were selected from each pen for the cutaneous basophil hypersensitivity (CBH) test using phytohaemagglutinin‐P. At 10 days of age, the first measurement of toe web thickness between the third and fourth digits of the right foot was done by a digital caliper (Xiaomi ATuMan CA2, 0.01‐mm precision). Then, 100 μg of phytohaemagglutinin (suspended in 0.10 mL of sterile saline) was injected into the toe web. The response was determined based on the difference between the first and the second measurement of the skin thickness at 24 and 48 h after injection (Kakhki et al., 2016).

### 2.6 White blood cells counting

At 42 days of age, 2 mL of blood was taken from the wing vein of the birds (two birds in each pen) and transferred into the tubes containing ethylene diamine tetra‐acetic acid. Blood samples were used to quantify the total leukocyte number by haemocytometer, using the Natt–Herricks solution (the samples were diluted with Natt–Herrick's solution in a ratio of 1:20). A drop of the solution containing white blood cells was poured in the centre of the haemacytometer slide, counting was done in nine large squares in the centre of the slide, and finally the number of white blood cells was expressed as a factor of 10^3^ per microliter of blood (Campbell, 1995). To determine the percentages of lymphocytes, monocytes, heterophils and heterophils to lymphocyte (H:L) ratio, blood smears were air‐dried, stained with Wright–Giemsa and counted using a light microscope (×1000 magnification).

### 2.7 Statistical analyses

This study was done based on a completely randomized design in a factorial arrangement of 4 × 2 with four levels of GP and two levels of MP with six replicates by the general linear model procedure of Statistical Analyses System (SAS) (2012) software. As the main effect of GP (quantitative factor) was significant, regression analysis was done. When the interactions were significant, the regression analysis for the GP within each MP (qualitative factor) level was performed, and their confidence intervals were compared. All data were normalized before statistical analysis. The main effect of MP and GP were compared by Tukey's test (*p* < 0.05).

## 3 RESULTS

### 3.1 Performance and internal organs

The effects of GP and MP supplementation on the growth performance of broilers are shown in Table 2. The effect of GP and MP on growth performance was not significant during the study (*p* > 0.05). Concerning the relative weight of immune organs, the main effects of GP on the relative weight of the bursa of Fabricius and thymus were significant (*p* < 0.05) (Table 3). According to the regression equation (Table 4), with increasing levels of GP in the diets, the relative weight of the mentioned organs increased. The non‐significance of lack of fit for both the relative weight of the bursa of Fabricius and thymus models indicated the excellent agreement of the data with their models (Table 4).

**TABLE 2:** Effects of different levels of garlic and mushroom powder on the performance of broilers during the starter, grower and finisher phases.

Treatments	Starter (1–10 days of age)			Grower (11–24 days of age)			Finisher (25–42 days of age)			Whole phase (1–42 days of age)		
Main effects	DWG (g/bird/days)	DFI (g/bird/days)	FCR (g:g)	DWG (g/bird/days)	DFI (g/bird/days)	FCR (g:g)	DWG (g/bird/days)	DFI (g/bird/days)	FCR (g:g)	DWG (g/bird/days)	DFI (g/bird/days)	FCR (g:g)
GP (%)												
0.00	21.88	27.23	1.25	48.59	71.41	1.47	79.84	129.78	1.63	55.62	85.90	1.54
0.50	22.12	27.99	1.26	48.18	71.57	1.49	78.16	125.72	1.61	54.82	84.41	1.53
1.00	22.32	28.11	1.27	48.17	71.36	1.48	78.94	127.36	1.62	55.20	85.13	1.54
1.50	22.29	27.09	1.23	48.21	71.98	1.52	79.37	129.22	1.62	55.41	86.24	1.56
*p* Value	0.89	0.28	0.62	0.77	0.52	0.51	0.66	0.54	0.95	0.52	0.61	0.91
SEM	0.449	0.441	0.022	0.328	0.875	0.024	0.973	2.169	0.029	0.385	1.038	0.018
MP (%)												
0.00	22.30	27.50	1.24	48.23	71.49	1.48	78.69	128.00	1.63	55.11	85.27	1.55
1.00	22.00	27.71	1.26	48.36	72.17	1.49	79.47	128.03	1.62	55.42	85.56	1.54
*p* Value	0.52	0.65	0.46	0.71	0.44	0.64	0.43	0.98	0.65	0.43	0.78	0.88
SEM	0.317	0.319	0.017	0.232	0.617	0.015	0.688	1.534	0.020	0.272	0.734	0.013
Interaction effects												
No GP + no MP	21.66	27.32	1.26	48.78	71.37	1.46	79.48	128.96	1.62	55.48	85.57	1.54
0.50% GP + no MP	22.81	28.30	1.24	47.34	70.98	1.50	76.30	122.53	1.61	53.91	82.91	1.53
1.00% GP + no MP	23.07	28.00	1.24	48.25	69.88	1.45	78.95	129.51	1.64	55.40	85.60	1.54
1.50% GP + no MP	21.67	26.38	1.22	48.56	73.74	1.52	80.03	131.03	1.64	55.64	87.01	1.56
No GP + 1.00% MP	22.09	27.12	1.23	48.40	71.44	1.48	80.21	130.60	1.63	55.77	86.24	1.55
0.50% GP + 1.00% MP	21.44	27.70	1.27	49.04	72.17	1.47	80.02	128.90	1.61	55.74	85.89	1.54
1.00% GP + 1.00% MP	21.58	28.22	1.30	48.12	72.85	1.51	78.93	125.22	1.59	55.00	84.67	1.54
1.50% GP + 1.00% MP	22.93	27.81	1.24	47.86	72.21	1.51	78.72	127.42	1.62	55.15	85.44	1.55
*p* Value	0.09	0.43	0.52	0.06	0.33	0.45	0.32	0.28	0.90	0.14	0.42	0.98
SEM	0.635	0.683	0.033	0.467	1.237	0.030	1.377	3.067	0.041	0.544	1.468	0.025

Abbreviations: DWG, daily weight gain; DFI, daily feed intake; FCR, Feed conversion ratio; GP, garlic powder; MP, mushroom powder; SEM, standard error of means.

**TABLE 3:** Effects of different levels of garlic and mushroom powder on relative organ weights (% of live body weight) in broilers at 42 days of age.

Main effects	Carcass yield	Liver	Spleen	Bursa of Fabricius	Thymus
GP (%)					
0.00	68.26	2.29	0.13	0.13	0.45
0.50	68.72	2.33	0.14	0.15	0.45
1.00	69.40	2.38	0.13	0.17	0.47
1.50	68.89	2.35	0.15	0.17	0.47
*p* Value	0.95	0.78	0.26	0.001	0.006
SEM	1.354	0.061	0.007	0.007	0.007
MP (%)					
0	68.12	2.34	0.14	0.15	0.46
1	69.51	2.32	0.14	0.16	0.45
*p* Value	0.32	0.92	0.91	0.65	0.30
SEM	0.981	0.043	0.005	0.005	0.005
Interaction effects					
no GP + no MP	67.23	2.24	0.12	0.13	0.44
0.50% GP + no MP	69.43	2.28	0.14	0.14	0.45
1.00% GP + no MP	68.15	2.27	0.13	0.17	0.47
1.50% GP + no MP	67.68	2.40	0.15	0.16	0.47
no GP + 1.00% MP	69.29	2.34	0.13	0.14	0.45
0.50% GP + 1.00% MP	68.00	2.37	0.13	0.15	0.45
1.00% GP + 1.00% MP	70.66	2.30	0.15	0.17	0.47
1.50% GP + 1.00% MP	70.09	2.35	0.14	0.16	0.48
*p* Value	0.70	0.50	0.35	0.74	0.92
SEM	2.098	0.089	0.010	0.009	0.010

Abbreviations: GP, garlic powder; MP, mushroom powder; SEM, standard error of means.

**TABLE 4:** Regression analysis of the main effect of garlic powder (quantitative factor) on the relative weight of bursa of Fabricius and thymus.

		Bursa of Fabricius			Thymus		
Source	df	Sum of squares	*R* ^2^	*p* Value	Sum of squares	*R* ^2^	*p* Value
Model	1	0.008	0.26	0.0002	0.007	0.23	0.0007
Lack of fit	2	0.001		0.2024	0.001		0.3887
Pure error	44	0.021			0.024		
Total error	46	0.023			0.025		
Equation		0.13 + 0.02 GP			0.4 + 0.02 GP		

Abbreviation: GP, garlic powder.

### 3.2 Estimation of humoural immune response

The effects of GP and MP on vaccination titres (NDV and AIV) are shown in Table 5. The interaction effects of GP and MP on the NDV titres were significant (*p* < 0.05). Analysis of regression of the effects of GP in each level of MP for NDV titre is presented in Table 6. The regression equations (Table 6) showed that the effect of increasing the amount of GP on the NDV titre was greater in the absence of MP. The lack of fit for both equations was insignificant, highlighting that the data were in good agreement with their model (Table 6).

**TABLE 5:** Effects of different levels of garlic and mushroom powder on humoural and cellular immunity responses in broiler chickens.

	Vaccination titres (42 days of age)		Hypersensitivity (mm)	
Main effects	NDV	AIV	After 24 h	After 48 h
GP (%)				
0.00	2.69	2.76	1.04	0.95
0.50	2.84	2.79	1.09	0.96
1.00	3.06	2.93	1.02	0.97
1.50	3.11	2.85	1.05	0.94
*p* Value	<0.0001	0.11	0.23	0.44
SEM	0.044	0.056	0.028	0.024
MP (%)				
0.00	2.94	2.87	1.11	0.98
1	2.91	2.81	1.06	0.96
*p* Value	0.50	0.27	0.18	0.36
SEM	0.031	0.040	0.020	0.017
Interaction effects				
No GP + no MP	2.61	2.71	1.04	0.95
0.50% GP + no MP	2.83	2.84	1.13	0.98
1.00% GP + no MP	3.09	2.97	1.16	1.03
1.50% GP + no MP	3.23	2.96	1.10	0.98
No GP + 1.00% MP	2.77	2.81	1.04	0.94
0.50% GP + 1.00% MP	2.85	2.75	1.05	0.95
1.00% GP + 1.00% MP	3.03	2.92	1.07	0.94
1.50% GP + 1.00% MP	2.99	2.75	1.07	1.01
*p* Value	0.02	0.30	0.42	0.46
SEM	0.063	0.079	0.040	0.034

Abbreviations: AIV, avian Influenza virus; GP, garlic powder; MP, mushroom powder; NDV, Newcastle disease virus; SEM, standard error of means.

**TABLE 6:** Regression analysis of the effects of garlic powder (GP) (quantitative factor) in each level of mushroom powder (MP) for Newcastle disease virus (NDV) titre.

		NDV titre					
		0 MP			1 MP		
Source	df	Sum of squares	*R* ^2^	*p* Value	Sum of squares	*R* ^2^	*p* Value
Model	1	1.365	0.68	<0.0001	0.232	0.33	0.0020
Lack of fit	2	0.023		0.6753	0.055		0.2427
Pure error	20	0.578			0.362		
Total error	22	0.601			0.650		
Equations		2.62 + 0.43 GP			2.78 + 0.18 GP		
Confidence interval		(0.30, 0.55)			(0.07, 0.28)		

### 3.3 Cutaneous basophil hypersensitivity

The data relating to CBH response are shown in Table 5. The CBH was not affected by different levels of GP and MP (*p* > 0.05).

### 3.4 White blood cells counting

The results of the white blood cell counts are given in Table 7. The main effects of GP on the percentages of lymphocytes and H:L ratio were significant (*p* < 0.05). According to the regression equation (Table 8), with increasing levels of GP in the diets, the percentages of lymphocytes and H:L ratio increased and decreased, respectively. The lack of fit for both of them was insignificant (Table 8), illustrating that the data fit well with the model.

**TABLE 7:** Effects of different levels of garlic and mushroom powder on white blood cell, and percentages of heterophils, lymphocytes, monocytes and heterophils to lymphocytes ratio in broiler chicken at 42 days of age.

Main effects	WBC (×10^3^/μL)	H (%)	L (%)	H:L ratio	M (%)
GP (%)					
0.00	27.23	26.36	61.33	0.43	8.27
0.50	27.38	26.09	63.34	0.41	8.30
1.00	27.73	26.48	64.97	0.40	8.97
1.50	27.65	26.10	65.03	0.40	8.53
*p* Value	0.23	0.77	0.003	0.01	0.26
SEM	0.192	0.339	0.616	0.006	0.287
MP (%)					
0	27.47	26.27	63.93	0.41	8.48
1	27.52	26.22	63.40	0.41	8.56
*p* Value	0.80	0.86	0.40	0.70	0.75
SEM	0.136	0.240	0.435	0.004	0.198
Interaction effects					
no GP + no MP	27.05	26.54	60.46	0.44	8.22
0.50% GP + no MP	27.20	25.70	64.18	0.40	8.21
1.00% GP + no MP	27.86	26.68	65.41	0.41	8.87
1.50% GP + no MP	27.78	26.19	65.66	0.40	8.60
no GP + 1.00% MP	27.40	26.18	62.20	0.42	8.32
0.50% GP + 1.00% MP	27.56	26.49	62.50	0.42	8.38
1.00% GP + 1.00% MP	27.61	26.28	64.53	0.41	9.07
1.50% GP + 1.00% MP	27.51	25.91	64.39	0.40	8.47
*p* Value	0.47	0.55	0.21	0.13	0.97
SEM	0.271	0.481	0.871	0.009	0.423

Abbreviations: GP, garlic powder; MP, mushroom powder; WBC, white blood cells; H, heterophils; L, lymphocytes; M, monocytes; SEM, standard error of means.

**TABLE 8:** Regression analysis of the main effect of garlic powder (GP, quantitative factor) on the percentage of lymphocytes and heterophils to lymphocytes ratio in broiler chicken at 42 days of age.

		Lymphocytes			Heterophils to lymphocytes ratio		
Source	df	Sum of squares	*R* ^2^	*p* Value	Sum of squares	*R* ^2^	*p* Value
Model	1	97.232	0.31	0.0001	0.005	0.19	0.0020
Lack of fit	2	12.323		0.2798	0.0005		0.5969
Pure error	44	206.776			0.021		
Total error	46	219.100			0.022		
Equation		61.76 + 2.55 GP			0.43 − 0.02 GP		

## 4 DISCUSSION

No significant difference was found in the mean DWG, feed intake (FI) and FCR during the experiment. In accordance with the present experiment, Choi et al. (2010) declared that GP had no considerable effects (0.00%, 1.00%, 3.00% and 5.00%) and α‐tocopherol on performance during the experiment. Overall, weight gain, FI and FCR in the control group were equal to other groups. It has been reported that body weight, FI and FCR were not affected by GP in laying quails (Yalcin et al., 2007). They suggested that the garlic's strong aroma does not work as a dissuasive of feeding. Aydogan et al. (2020) did not discover significant differences between treatments when using different garlic and black cumin levels in broiler diets. In contrast, Borgohain et al. (2017) observed that GP at the rate of 0.50%, 1.00% and 1.50% increased FI and improved FCR in the broilers receiving diets containing garlic. Varmaghany et al. (2015) expressed that the final live body weight and weight gain at 1–42 days of age mounted with increasing garlic bulb levels in the diets. Feeding garlic at the greatest level (15.00 g/kg) diminished final body weight and 1–42 days weight gain. The FI was linearly decreased with increasing garlic bulb levels in the diets at 1–42 days. These differences in studies may be due to several factors such as the amount of garlic used, the diets’ composition and the agricultural situation. Lima et al. (2021) stated that *Agaricus subrufescens* and *Pleurotus ostreatus* mushrooms could be used in broilers’ diets without compromising intestinal or haematological situations. However, these ingredients did not eventuate in improvements in growth performance. Guo et al. (2003) declared the great variety of the physicochemical attributes of different mushroom polysaccharides, such as sugar composition, molar weights and structures. These diversities cause different results.

The data from the current study showed that the relative weights of the bursa of Fabricius and the thymus were affected by GP. It has been reported that the increase in the spleen, thymus and bursa of Fabricius size due to the consumption of different levels of garlic in the diet, probably due to the stimulation of the proliferation of lymphocytes and the increase in the number of white blood cells (Hanieh et al., 2010). A greater weight of the bursa of Fabricius generally indicates a better anatomical response and premiere health in the face of alteration in immunity because of stress (Willis et al., 2007). In agreement with our study, Rahimi et al. (2011) reported that dietary garlic significantly increased the relative weight of bursa of Fabricius compared with the control diet. It has been shown that the spleen and thymus relative weights were higher in broilers receiving diets with 200 mg/kg star anise oil than those of the control group (Ding et al., 2020). Aziz‐Aliabadi et al. (2023) declared that different levels of green tea and mulberry leaves powder had no significant effect on the immune organ's weight in broilers. Sunu et al. (2021) observed that the weight of the thymus and bursa in treatments containing synbiotics was remarkably greater than the control. In contrast, Cho et al. (2014) and Amad et al. (2011) reported no difference in the relative weight of organs in broiler chickens’ nourished essential oils derived from thyme and star anise. Lee et al. (2016) stated that the relative weight of the liver, spleen, bursa of Fabricius and abdominal fat was not affected by different levels of fermented garlic. In agreement with the current study, many studies have reported that mushrooms did not affect lymphoid organ weights (Attia & Al‐Harthi, 2015; Hassan et al., 2020; Mahfuz et al., 2018; Shamsi et al., 2015).

Natural antioxidants maintain the health of animals and humans by regulating immune routes that adjust and improve the detrimental traces of inordinate generation of reactive oxygen species (Puertollano et al., 2011). In the current trial, the NDV titre increased with the increase of GP level in the diets, and the use of MP did not significantly affect the vaccine titre. In agreement with our results, Toghyani et al. (2012) also did not report a substantial dissimilitude in terms of NDV and AIV titres between oyster MP‐consuming groups and the control. Daneshmand et al. (2011) reported that the presence of oyster mushrooms in the diets of broiler chickens reduced the NDV titre at 21 days of age. The amount of MP inclusion in the diets may affect their lack of statistically significant effects on immune responses. The levels of MP in the present study were probably not optimal for improving immune responses in broilers. Hayat et al. (2022) stated that in broilers consuming 0.6% of GP, the titre of the Newcastle vaccine was significantly higher than the control group. It has been reported that immune‐modulating substances in garlic can improve the birds’ immune response and reduce the loss of the body's defence mechanism due to ageing (Rehman & Munir, 2015). In agreement with the present study, Hassan et al. (2013) confirmed that antibody titre against the Newcastle virus improved in birds consuming garlic extract. It has been observed that greater antibody titres against Newcastle and influenza in broilers consumed garlic and garlic‐aloe vera in different ratios (Fallah, 2014). It has been suggested that the garlic stimulatory impacts on humoural immunity may be due to the improved immune cell tasks that produce cytokine and/or phagocytic capacity of antigen‐presenting cells (Hanieh et al., 2010). In contrast, Jafari et al. (2008) stated that the inclusion of 1% and 3% of GP in the diet did not affect the serological response of broilers to the Newcastle vaccine. Toghyani et al. (2011) also did not observe any significant difference between treatments regarding immune responses when using different levels of cinnamon and garlic in broiler diets.

In the present experiment, different levels of GP and MP did not affect the CBH response. On the other hand, Oladele et al. (2015) reported that feeding garlic to broiler chickens increases the delayed reaction of the footpad, which is a sign of stimulated cellular immunity. Kamboh et al. (2018) showed that using genistein or hesperidin in the diet remarkably raised the CBH response of broilers. The reason for these various responses can be the experimental and management conditions, the birds’ physiological status, stress and diet quality.

The H:L ratio has been recognized as an index to consider the level of stress in poultry, so the decrement in this ratio represents the birds’ ability to manage stress better (Gross & Siegel, 1983). Heterophils (which in birds are tantamount to neutrophils in mammals) are a sector of the innate immune system. Thus, the decreased H:L ratio can be explained as amplifying the acquired immune system (Humphrey & Klasing, 2004). In the current study, according to the regression equation obtained with the enhancement of the level of GP in the diet, the H:L ratio was reduced. In agreement with our results, Mohammadi Motamedi and Mehdizade Taklolmi (2014) stated that garlic decreased the H:L ratio. It has been reported that the presence of garlic in pigs’ diets increases the concentration of lymphocytes in the blood. The boosted lymphocyte duplication by garlic, along with the plausible defence of the cells from oxidative stress, seemed to contribute to the increased white blood cell count reported in their study (Chen et al., 2008). Some studies have shown that sulphur substances in garlic have the feature of regulating the immune system, and garlic extract enhances the growth and development of lymphocytes in mice (Windich et al., 2008). In accordance with the current study, Ismail et al. (2021) reported that lymphocytes and heterocysts levels increased with GP and phenylacetic acid supplementation as compared to the control. Eid and Iraqi (2014) also stated that garlic increased the white blood cells, heterophils, lymphocytes and H:L ratio and ascribed it to a stimulatory effect of garlic on the immune response. Elagib et al. (2013) realized that diets comprising 3% and 5% GP had no significant effect on the number of white blood cells in broilers. It has been shown that there was no treatment effect for immune cell counts (heterophils, lymphocytes) at 21 days of age (Lima et al., 2021). The study of Fadlalla et al. (2010) highlighted that 0.3% garlic significantly improved the cellular immune response of broilers via enhancing total white blood cell count at the end of the study. Guo et al. (2003) declared that under infectious states, the effects of mushrooms showed more than normal ones.

## 5 CONCLUSIONS

The results of this study showed that increasing the level of GP improved the immune response of broilers without affecting performance. The use of MP did not affect the measured traits. With increasing levels of GP, the titre of the Newcastle vaccine was higher in the absence of MP compared to the presence of MP.

## AUTHOR CONTRIBUTIONS

*Conceptualization; formal analysis; data curation; funding acquisition; investigation; methodology; project administration; resources; software; writing – original draft; writing – review and editing*: Hadi Noruzi. *Conceptualization; formal analysis; funding acquisition; investigation; methodology; project administration; resources; software; writing – original draft; writing – review and editing*: Fatemeh Aziz‐Aliabadi.

## CONFLICT OF INTEREST STATEMENT

The authors declare no conflicts of interest.

## FUNDING INFORMATION

All the costs of the experiment were covered by the authors’ personal funds.

### PEER REVIEW

The peer review history for this article is available at https://publons.com/publon/10.1002/vms3.1367.

## ETHICS STATEMENT

All procedures were approved by the Animal Care and Use Committee of the Ferdowsi University of Mashhad, Mashhad, Iran.

## AUTHOR DECLARATIONS

All authors are either employed by or associated with, a government agency or university, whose primary function is research and education.

## Data Availability

The data that support the findings of this study are available from the corresponding author upon reasonable request.
