# Expression of Concern: Garlic (*Allium sativum*) and mushroom (*Agaricus bisporus*) powder: Investigation of performance, immune organs and humoural and cellular immune response in broilers

**DOI:** 10.1002/vms3.1541

**Published:** 2024-07-04

## Abstract

H. Noruzi and F. Aziz‐Aliabadi, “Garlic (*Allium Sativum*) and Mushroom (*Agaricus Bisporus*) Powder: Investigation of Performance, Immune Organs and Humoural and Cellular Immune Response in Broilers,” *Veterinary Medicine and Science* 10, no. 2 (2024): e31367, https://doi.org/10.1002/vms3.1367.

This Expression of Concern is for the above article, published online on 15 February 2024 in Wiley Online Library (wileyonlinelibrary.com), and has been published by agreement between the journal Editor‐in‐Chief, Gayle Hallowell and John Wiley & Sons Ltd. The Expression of Concern has been agreed due to concerns raised by a third party regarding the availability of an ethical approval. The authors have received Higher Degree by Research (HDR) committee approval and a bioethical course certificate. The authors and their institute confirmed that this was equivalent to an ethical approval from the Ferdowsi University of Mashhad at the time when the research was conducted but could not provide the HDR committee approval documentation. Since this does not fully comply with the ethics policy of the journal, as noted on the journal's author guidelines page, the journal has decided to issue an Expression of Concern to inform and alert the readers.

